# Absence of Adverse Effects on Pulmonary Histopathology and Functions Following Inhalation Exposure to Chloromethylisothiazolinone/Methylisothiazolinone

**DOI:** 10.3390/toxics13060482

**Published:** 2025-06-06

**Authors:** Sam Kacew, Esref Demir

**Affiliations:** 1 McLauglin Centre for Population Health Risk Assessment, University of Ottawa, Ottawa, ON K1N 6N5, Canada; 2 Medical Laboratory Techniques Program, Department of Medical Services and Techniques, Vocational School of Health Services, Antalya Bilim University, Antalya 07190, Türkiye

**Keywords:** chloromethylisothiazolinone, methylisothiazolinone, humidifier disinfectant, Kathon, inhalation, fibrosis, lung toxicity

## Abstract

In South Korea, issues have been raised regarding exposure to humidifier disinfectant products containing certain chemicals postulated to induce lung diseases in consumers. Several rodent studies utilizing whole-body inhalation, which comprises freely moving animals breathing through the nares, and intranasal instillation involving restraint, were conducted by various Korean Governmental Agencies on these products to investigate whether there is a causal relationship between these products and the development of lung diseases. In particular, the humidifier disinfectant product Kathon, containing chloromethylisothiazolinone and methylisothiazolinone (CMIT and MIT), when directly introduced into inhalation chambers at varying concentrations for up to 13 weeks, produced no significant histopathological alterations and no marked changes in pulmonary function parameters. Further, there was no evidence of cytotoxicity; total and differential cell counts did not differ from control. In addition, the levels of cytokine markers of inflammation were not markedly altered. In contrast to published papers utilizing intratracheal and intranasal instillation, where the animal is anesthetized and chemical bypasses the defense mechanisms in the respiratory tract, then reaches the pulmonary region, ignoring recommended dose levels was found to initiate fibrotic responses in mice and rats. However, the usefulness of experimental results to extrapolate to humans obtained following intratracheal and intranasal instillation studies is of limited value because the data generated did not use a realistic design and appropriate dosimetry. Therefore, these findings have significant drawbacks in their use to characterize an inhalation risk for pulmonary fibrosis in humans and cannot be used for the extrapolation of such risk to humans. It is thus evident that the inhalation data generated by the Korean Regulatory Agencies are more realistic and show that exposure to CMIT and MIT does not initiate pulmonary fibrosis. Although inhalation studies still do not fully replicate real-world human exposure scenarios and have limitations for direct extrapolation to humans, they are nevertheless more appropriate than intratracheal or intranasal instillation models.

## 1. Introduction

The association between exposure to humidifier disinfectant (HD), containing a mixture of chloromethylisohiazolinone (CMIT) and methylisothiazolinone (MIT) brand name (Kathon CG), and damage to the lung is controversial based on the experimental protocol and route of exposure. Several investigators reported that HD containing CMIT and MIT initiated serious lung damage following intratracheal instillation of Kathon 1.14 mg/kg to mice, as evidenced by an elevated proportion of macrophages, eosinophils, and neutrophils [1]. In addition, Song et al. [1] noted that Kathon administered intratracheally, produced histopathological increases in perivascular/alveolar inflammation, mucous cell hyperplasia, and pulmonary fibrosis. In a subsequent experiment, Song et al. [2] compared intratracheal instillation of 2 different concentrations of Kathon 0.57 and 1.14 mg/kg which is a mixture of CMIT and MIT, and compared these observations to intranasal 1.14 mg/kg intranasal instillation in mice. Song et al. [2] showed that following intratracheal instillation the total number and pathology scoring of granulomatous inflammation/pulmonary fibrosis as well as hyperplasia in mucous cells, bronchi, and bronchioles was detected with a dose of CMIT and MIT 0.29 mg/kg and these observations increased in severity maximally at 0.57 and 1.14 mg/kg CMIT and MIT. In contrast, intranasal instillation of 1.14 mg/kg in mice produced no marked effect on granulomatous inflammation/pulmonary fibrosis and hyperplasia in mucous cells, bronchi, and bronchioles [2].

It is important to note that these published papers [1,2] acknowledge shortcomings in experimental design and execution in intratracheal instillation [3,4]. These include non-physiological exposures involving termed “bolus effect” [3,4] where high CMIT and MIT doses are placed directly in contact with lung tissue; however, the serious problem which limits intratracheal instillation is that the distribution of dose to the respiratory tissues is highly artificial [5]) and does not reflect accurately lung deposition patterns of chemical following inhalation exposure. Intratracheal instillation creates an artifactual series of cellular (macrophage reactions, such as inflammation and fibrosis), which do not reflect the events that occur following inhalation exposure [3]. With intratracheal instillation, if the amount of deposited material in the lung exceeds a certain limit, the pulmonary responses are influenced not only by, for example, CMIT and MIT but also by the phenomenon of “overload,” that is, surplus dose [6,7]. This overload of chemicals leads to disturbance in the elimination of materials from the lung and may be associated with severe inflammation and fibrotic responses [8]. It is important to note that inhalation of nanomaterials, compared to intratracheal instillation in rats, showed that intratracheal instillation initiated pulmonary inflammation, but inhalation did not induce any adverse effects in the lung. Further, Morimoto et al. [9] reported that intratracheal instillation of high-dose fullerenes induces pulmonary inflammation in rats, whereas inhalation of this compound produced no effects in lung tissue. These findings clearly demonstrate that by placing the chemical directly into lung tissue by intratracheal instillation, this experimental procedure either directly induces inflammatory, fibrotic responses or indirectly, by “chemical overload,” initiates damage.

Although intranasal instillation initiated a diminished frequency and severity of pulmonary injuries detected following CMIT and MIT administration [2], the utility of these findings to be extrapolated to humans exhibits limitations including verification of administered dose to the lung, limited species with respect to physiologic and anatomical characteristics that are relevant for a proper, accurate hazard characterization, anesthetization and restraint of animals to prevent normal defense clearance mechanisms and bypass of protective enzymes involved in degradation and elimination in the respiratory tract [10,11,12,13,14].

It is thus evident that the methodologies utilized to determine the effects of CMIT and MIT on humans play a critical role in the consequences reported in the lungs. Driscoll et al. [5] noted that evaluation of respiratory tract toxicity from airborne materials involves exposure of animals via inhalation, which is the “natural route of entry of substances” into the host and is the “preferred” method for the introduction of toxicants into the lungs. In order to confirm the realistic use of experimental protocols suitable for risk assessment and correlation to humans, the methodologies should be extended to properly designed inhalation studies, perhaps in multiple relevant species, towards defining a risk assessment. Using properly designed inhalation studies in multiple species, combined with appropriate epidemiology and exposure data, a proper risk assessment may be performed using a weight-of-evidence approach. As indicated by Kim et al. [15], at present, there is an “insufficient body of papers reporting inhalation exposure and accordingly respiratory health effects in peer-reviewed outlets, Government documentation, and official but not peer-reviewed research reports”. Thus, the aim of this study was to present the experiments conducted by several Korean Governmental Agencies which conducted inhalation studies with CMIT and MIT.

## 2. Experimental Protocol and Results

These studies were conducted by several (1) Korean Government Agencies, in particular, the Korean Disease Control and Prevention Agency (KCDA) in 2012; (2) airborne exposure assessment study was conducted by the Ministry of Environment (MOE) in 2019, (3) Korean Environmental Industry& Technology Institute (KEITI) in 2017, (4) Safety Evaluation Research Institute, a subsidiary of the Korean Research Institute of Chemical Technology, published by the National Institute of Environmental Research (NIER) in 2018, (5) Korea Institute of Toxicology (KIT) in 2022, and (6) Korea Institute of Toxicology (KIT) in 2023. In all studies, the relative humidity (50%: +10%) and under controlled temperature (22 + 3 °C) with a 12 h light/dark cycle.

### 2.1. Korean Disease Control and Prevention Agency (KCDA) Study

This airborne exposure assessment and full-body inhalation exposure study was conducted by the Korean Disease Control and Prevention Agency (KCDA) in 2012.

Airborne concentrations of Humidifier Mate were measured following dispersion using an ultrasonic humidifier (NHU-5502C0 at 1-fold, 2-fold, or 10-fold higher than the recommended usage (RU) in a 47.3 m^3^ space with a ventilation rate of 1 air exchange/h. The chemical was dispersed continuously over an 8 h duration. Data demonstrated that the compound was not detected at 1- or 2-fold RU, whereas at the 10-fold RU, the concentration was 0.013–0.015 mg/m^3^. When converted to mass concentration, rats were exposed to 1.83 mg/kg.

Sprague-Dawley rats were purchased from Orient Bio Inc. The control animals received only air. Forty male rats weighing approximately 190 g and 40 female rats weighing approximately160 g were placed in a whole-body inhalation toxicity chamber (HCT, Republic of Korea) sand exposed to Humidifier Mate (SK Chemical Inc., Seongnam-si, Republic of Korea) at a concentration of 1.83 mg/m^3^ (10-fold higher than RU) for 6 h/day for 5 times/week for 13 weeks. After the exposure period rats were anesthetized with pentobarbital the next day and examined for histopathological testing.

There was no evidence of histopathological pulmonary injury as the morphology and cells appeared similar to the control. Further, with the use of cytokine kits, the levels of IL-1 β and Il-6 were not significantly different in males and females compared to controls, indicating the absence of an inflammatory response. In addition, the measurement of TGF β, biomarkers of fibrosis, was not markedly different from controls in males. In the 13-week exposure group, a 2.8-fold rise was noted in females compared to controls. However, no significant alterations were detected in other parameters. These findings clearly demonstrated that whole-body exposure to Humidifier Mate (CMIT and MIT) did not induce lung damage in rats.

### 2.2. Ministry of Environment (MOE) Study

This airborne exposure assessment study was conducted by the Ministry of Environment (MOE) in 2019.

Further to the KCDA study in 2012, a more precise methodology was employed to measure airborne levels of CMIT and MIT. Experiments were conducted in a 30 m^3^ chamber at concentrations of 1-, 5-, and 10-fold higher than RU, as well as ventilation rates of 0.3, 0.5, or 1 air changes/h. The compound was dispersed over a cumulative exposure period of 6, 9, or 13 h. At 1-fold RU, the measured concentration ranged from 0.0011 to 0.0013 mg/m^3,^ similar to the KCDA study.

### 2.3. Korean Environmental Industry & Technology Institute (KEITI) Study

This full-body inhalation exposure study was conducted by the Korean Environmental Industry & Technology Institute (KEITI) in 2017.

In this in vivo Phase 1 investigation, a full-body inhalation exposure was conducted for 8 sessions. Forty Balb/c mice with 10 animals/group were exposed to Kathon CG (CMIT and MIT 1%) at exposure concentrations of either 1, 70, or 150 mg/m^3^ (5.5, 388, and 833-fold higher than RU) for 2 h/day at 5 times/week for 2 weeks using a Constant Atomizer Type Control generator. Control mice were subjected to air only.

The parameters examined included organ weights, body weight, pulmonary function tests, and biomarkers measured in serum were total and differential cell counts and immunoglobulin E (IgE) levels. Further bronchoalveolar lavage fluid (BALF) was obtained to determine lactate dehydrogenase (LDH) activity.

Data demonstrated that whole-body inhalation of CMIT and MIT did not markedly alter airway hyperresponsiveness or allergic reactions. Further, there was the absence of airway mucosal inflammation as evidenced by no significant alterations in cytokine levels, lack of immune system compromise as found by no effects on total and differential cell counts, and no evidence of cytotoxicity or pulmonary diseases as noted by no significant change in LDH levels. These findings showed that whole-body inhalation exposure in mice to CMIT and MIT did not markedly affect pulmonary function and biochemical parameters.

### 2.4. Korean Research Institute of Chemical Technology Study

This full-body inhalation exposure study was conducted at the Safety Evaluation Research Institute, a subsidiary of the Korean Research Institute of Chemical Technology published by the National Institute of Environmental Research (NIER) in 2018.

Twenty male Wistar/CrjOri:Wistar rats and 20 female Wistar/CrjOri:Wistar rats were exposed to a Mist generator (NB-2N, Sibata) containing Kathon CG Preservative (CMIT 1.124%, MIT 0.368%) at a concentration of 25 or 50 mg/m^3^ (138 and 277-fold higher than RU) for 20 h/day 7 times/week, for 4 weeks. Control rats were subjected to air only.

During the full-body exposure, animals were monitored for body weight and feed consumption with no marked alterations from the control. At the termination of exposure, rats were anesthetized with pentobarbital the following day, and blood was obtained by cardiac puncture to determine the total and differential cell count. A portion of the blood was centrifuged at 3000 rpm to obtain the serum for measurement of IgE. A portion of the lungs was perfused with saline to obtain the BALF for measurement of LDH activity. The unlavaged left lung was inflated with 10% neutral buffered formalin (NBF), embedded in paraffin, cut at 5 µm on slides, and stained with Hematoxylin and Eosin (H&E) for histopathological evaluation.

The results from the Safety Evaluation Research Institute, a subsidiary of the Korean Research Institute of Chemical Technology published by the National Institute of Environmental Research (NIER) in 2018 showed that exposure to CMIT1.124%/MIT 0.368% did not exhibit any signs of pulmonary inflammation or fibrosis as evidence by no marked changes in serum cytokine IgE levels, total and differential blood cell counts, absence of cytotoxicity as determined by LDH activity levels and importantly no marked histopathological alterations in lung tissue.

### 2.5. Korea Institute of Toxicology (KIT) Study

This full-body inhalation exposure study was conducted by the Korea Institute of Toxicology (KIT) in 2022.

The C57BL/6 mice were purchased from Orient Bio, Inc. (Korea). A total of 42 male mice were exposed to Kathon CG Preservative (CMIT 1.124%/MIT 0.368%) in an Inhalation Chamber (KIT-A0192004-0040) at concentrations of either 5 or 15 mg/m^3^ (27 and 83-fold higher than RU) for 2 h/day, 3 or 5 times a week for 2 weeks. Control mice were subjected to air only.

The C57BL/6 mice were anesthetized with isoflurane. Body weight was recorded, blood was obtained by cardiac puncture, and tissues were extracted and weighed at day 15. A portion of the lungs was perfused with saline to obtain the BALF for total cell count and differential cell count assessment. The unlavaged left lung was inflated with 10% neutral buffered formalin (NBF), embedded in paraffin, cut at 5 µm on slides, and stained with Hematoxylin and Eosin (H&E) for histopathological evaluation.

Data showed that the 5 mg/m^3^ (27-fold higher than RU) concentration of CMIT and MIT produced no significant alterations in total and differential cell counts, BALF cytokine levels, and no changes in lung histopathology. Although a significant increase in inflammatory cells, macrophages, and neutrophils in BALF total cell count, as well as elevated levels of cytokines including IL-4, IL-17, TNF-α, MCP-1, IL-1β, KC/CXCL1, and IL-6, was detected only in the 15 mg/m^3^ (83-fold higher than RU) concentration of CMIT and MIT group exposed five times per week, this finding was not associated with any pulmonary pathological changes indicating there was no evidence of pulmonary fibrosis.

### 2.6. Korea Institute of Toxicology Study

This nose-only inhalation exposure study was conducted by the Korea Institute of Toxicology (KIT) in 2023.

The C57BL/6 mice were purchased from Orient Bio, Inc. (Korea). A total of 40 male mice were exposed to Kathon CG Preservative (CMIT 1.124%/MIT 0.368%) in an Inhalation Chamber (VITALS, KIT-A0207033) with a distance between the mist and the generator and the nasal chamber set to less than 50 cm. The concentration of CMIT and MIT was 0.75, 1.5, or 3 mg/m^3^ (4.7-, 8.3-and 16-fold higher than RU) for 2 h/day, 5 times a week for 2 weeks. Control mice were subjected to air only.

The C57BL/6 mice were anesthetized with isoflurane. Body weight was recorded, blood was obtained by cardiac puncture, and tissues were extracted and weighed. A portion of the lungs was perfused with saline to obtain the BALF for the measurement of total cell counts and differential cell counts. The unlavaged lung was inflated with 10% neutral buffered formalin (NBF), embedded in paraffin, cut at 5 µm on slides, and stained with hematoxylin and eosin (H&E) for histopathological evaluation. A portion of the right lung lobe was employed to measure changes in the expression of damage-related proteins in the tissue.

At 0.75 or 1.5 mg/m^3^ of CMIT and MIT, equivalent to 4.7 or 8.3-fold higher than the recommended usage (RU), there was no evidence of histopathological alterations in the lung. These observations were associated with no marked alterations in tissue weight, serum total and differential cell counts, pulmonary cytokine, and fibronectin levels, as well as LDH activity in BALF. However, exposure to CMIT and MIT at 3 mg/m^3^ equivalent to 16.6-fold higher RU the following parameters were noted: increased lung weight and tissue discoloration, pulmonary hemorrhage, elevated levels of Th-2 associated cytokines (IL-4, IL-5, IL-13, IL-17, IL-1β, IL-6, TNF α, and MCP-1), epithelial cell damage markers (TSLP, IL-25 and IL-33) as well as fibrosis biomarker (fibronectin). Thus, the no-observed effect concentration (NOEC) for CMIT and MIT on pulmonary morphology and biomarkers of tissue injury was 1.5 mg/m^3^ of humidifier disinfectant, equivalent to 8.3-fold higher than the recommended use.

Although the degradation products of CNIT and MIT were not measured, our findings show that exposure to parent CMIT and MIT or potential metabolites did not induce pulmonary fibrosis.

## 3. Discussion

Exposure to humidifier disinfectant (HD) containing a mixture of chloromethylisohiazolinone (CMIT) and methylisothiazolinone (MIT), brand name (Kathon CG), and lung injury was previously based upon data generated by intratracheal instillation, in particular the studies of Song et al. [1,2]. These investigators indicated that following intratracheal instillation of Kathon, which is a mixture of CMIT and MIT, at doses of 0.57 or 1.14 mg/kg induced fibrotic responses in the mouse lung. Song et al. (1) utilized exposure to Kathon administered intratracheally at a dose of 0.57 mg/kg or 1,14 mg/kg. The authors did not provide information on concentrations of CMIT and MIT within the dose of 0.57 mg/kg or 1.14 mg/kg Kathon, but that exposure was to the mixture at these doses. These observations led to the conclusion that Kathon “may help patients who complain of fibrosis are recognized as victims and help determine potential victims” However, this conclusion is flawed, based upon the distribution of dose to the respiratory tissues which is highly artificial, does not reflect accurately lung deposition patterns of chemical following inhalation exposure [3] and thus data generated can NOT be extrapolated to humans as indicated below. In order to understand the experimental protocol and why it cannot be applied to humans, one needs to understand the functions of the upper airway in the human lung. Further, it is critical to understand that in order to carry out intratracheal and intranasal instillation, the animal is not freely moving and must be anesthetized, which indicates that the normal clearance functions of the nasopharyngeal and tracheobronchial (TB) regions are inhibited [11,16,17].

When a human individual is exposed to particles or contaminants, such as Kathon, defense mechanisms in the nasal and TB region, including mucociliary clearance, consist of a protective mucous layer and cilia that propel inhaled contaminants out of the airways [18]. The mucociliary clearance mechanism is critical for the lung to defend against Kathon, but this function is absent in the intratracheal instillation model. It should be noted that mucociliary clearance is interfered with following intranasal delivery of substances altering absorption and the amount of chemical reaching the lung [18,19]. Thus, the use of intratracheal and intranasal instillation does not simulate the human condition and should not reflect “fibrosis-related responses” in humans.

### 3.1. Metabolism

Environmental agents may enter the lung via the nasal or TB tree [20]. The nasal mucosa contains xenobiotic metabolic enzymes, including cytochrome P450 monooxygenases, aldehyde dehydrogenases, epoxide hydrolases, carboxyl esterases, and glutathione transferases to protect against damage to the respiratory system [21,22]. In the TB airways, epithelial cells contain cytochrome P450, in particular CYP2E1, to degrade environmental agents [23]. This xenobiotic metabolic enzyme mechanism system becomes activated once the chemical enters the nose and TB tree and serves to destroy the contaminant [12,23]. In intratracheal and intranasal instillation, this metabolic system is bypassed [3,10,24] and hence does not reflect extrapolation to humans. The xenobiotic system becomes activated upon chemical exposure.

### 3.2. Clearance

The TB region, which consists of the trachea and bronchial tree down to the terminal bronchioles, functions to deliver inhaled air, which may contain contaminants [16]. The primary clearance mechanism for this TB region is similar to the nasal clearance system. In the TB region, you also have mucociliary clearance, where you have ciliated and secreting cells to remove deposited material [25]. The airway epithelium is capable of secreting and releasing mucus, ions, and water needed to remove unwanted chemicals [25]. In intratracheal and intranasal instillation, where you require anesthesia [17], this TB clearance functioning system is not active and hence does not simulate extrapolation to humans.

The paper by Song et al. [1] (p. 12, para. 2.) itself explicitly acknowledges these limitations: “The current study has several limitations. First, intratracheal instillation is not a satisfactory alternative to inhalation and may result in markedly different distribution, transport, and toxicity of the test substances in the lungs.” The Song et al. [1] paper acknowledges that instillation may serve only as a preliminary experiment, suggesting the potential effects of excessive amounts of chemicals reaching the deep lung. However, it should be kept in mind that these findings do not reflect realistic environmental inhalation toxicity in humans [15].

It is important to note that when mucociliary clearance is absent or not functioning due to the anesthetic state as in the case of intratracheal instillation the concentration detected in the lung is excessively high as the contaminant is delivered as a “bolus” dose which does not reflect the actual human exposure amount and dose rate [3]. Specifically, bolus doses of 0.57 mg/kg and 1.14 mg/kg were administered resulting in contaminant overload [4,26], where are much higher, both concentration and dose rate are much higher than what consumers would have been exposed to when using Humidifier Mate according to the product instructions.

### 3.3. Intratracheal Instillation Drawbacks

In human’s nasal and TB metabolic and physiological mechanisms are activated to deal with contaminant utilization clearance, metabolism, antioxidants, and lung mechanics to protect the lung from injury [20,21,22,23]. Intratracheal instillation has inherent drawbacks, including the need for strong anesthetics and survival surgery [26]. Bolus dosing of such high concentrations can induce toxicity in animals not due to any intrinsic toxicity of the substance itself but because the substance is administered too much or too quickly [3]. Delivery via intratracheal instillation causes overload in rats and is not equivalent to human inhalation exposure [27]. Further, according to the Korean Disease Control and Prevention Agency (KCDA) in 2012, exposure to a 10-fold higher concentration than RU to CMIT and MIT showed no evidence of lung damage or fibrosis. The toxicity of the substance itself, administered under these conditions, may not be representative of inhalation of a distributed material in which exposure occurs over a longer period of time at much lower doses and dose rates [27]. Bolus dosing of high doses in an extremely short time overwhelms the body’s defense mechanism and induces artifactual injury [3,4]. Further, the intratracheal instillation regimen produced the early hallmark of acute lung injury as evidenced by inflammatory responses unrelated to the environmental agent [4,28,29]. The mechanism and distribution pattern that is seen from such dosing is completely different from, and not representative of, how the body actually reacts in a real-world situation [30]. Thus, selecting an appropriate dosing concentration and rate is critical, as even a benign substance might exert a toxic effect if the substance is not dosed properly, as evidenced by overload inflammatory reactions [4]. While bolus-type dosing may be used in a preliminary experiment of a substance, it needs to be followed by a realistic inhalation study with environmentally relevant dosing to examine the consequences following xenobiotic inhalation exposure. Both intratracheal and intranasal instillation studies, however, do not allow an understanding of how the results can be contextualized in relation to the inhalation risk in humans. Particularly, the intratracheal instillation study, with its stated limitations, is designed as an initial proof of concept. However, limitations of intratracheal and intranasal findings [4,11,14,31] leave significant uncertainties in their extrapolation to human risk, and data must be used cautiously prior to deriving any conclusions.

### 3.4. Limitations

Although the technique of intratracheal and intranasal instillation is not expensive [13], high concentrations of the test agent Kathon (CMIT and MIT) can be placed directly in contact with the lung tissue, this serious problem limits the usefulness of intratracheal and intranasal instillation is that the distribution of dose to the respiratory tissue is highly artifactual and does NOT accurately reflect lung distribution of chemical following inhalation exposures [3,10,12].

As an example, with intratracheal instillation of 1.14 mg/kg Kathon, the concentration of [^14^C]CMIT and MIT was 2720 ng/g while with intranasal exposure the concentration of [^14^C]CMIT and MIT was 752 ng/g which was 3-fold lower but the important point is that with intratracheal instillation slight to moderate lung fibrosis was detected but no fibrosis with intranasal inhalation [2]. This again indicated that the concentration of Kathon delivered to lung tissue is critical for an effect.

As shown Korea Institute of Toxicology (KIT) in a 2023 study, exposure to 4.7-fold higher than RU. It should be noted that 8.3-fold higher than the RU to CMIT and MIT produced no evidence of lung injury and fibrosis. Further, according to the Korean Disease Control and Prevention Agency (KCDA) in 2012, exposure to 10-fold higher than RU to CMIT and MIT RU, no evidence of lung damage and fibrosis was detected. However, when the RU was raised to 16.6-fold higher, lung discoloration and hemorrhage were accompanied by inflammation and fibrosis, indicating that the concentration of CMIT and MIT reaching the lung is critical. Under realistic environmental conditions, humans are not subjected to 16.6-fold higher CMIT and MIT compared to RU. In essence, intratracheal instillation resulted in pulmonary fibrosis, whereas under inhalation exposure, there was no evidence of pulmonary fibrosis, inflammation, or injury, as well as no evidence at concentrations ranging from at least 4.7-fold to a maximum of 10-fold higher in dose than RU. It is important to note that the US EPA exposed rats to inhalation of CMIT and MIT in a 13-week inhalation study and found no evidence of systemic toxicity [32]. In essence, intratracheal instillation resulted in pulmonary fibrosis, whereas under inhalation exposure, it produced no evidence of lung injury at concentrations ranging from at least 4.7-fold to a maximum of 10-fold higher in dose than RU.

## 4. Conclusions

In summary, the use of intratracheal and intranasal instillation, which requires anesthesia, enables a bolus administration of Kathon to be placed in the lung or nose to subsequently induce fibrosis; whereas an inhalation toxicity study that more closely resembles human exposure did not induce pulmonary fibrosis. As indicated by Kim et al. [15], it is essential to note that “there is insufficient evidence in all related areas, including inhalation exposure assessment studies” regarding the effects of exposure to CMIT and MIT on lung function and parameters. The conclusion is that Kathon-induced fibrotic responses are merely an experimental observation based upon intratracheal and intranasal instillation, experimental protocols that exhibit limitations and are flawed with respect to extrapolation to humans. Evidence is provided for inhalation exposure assessment that clearly demonstrates that exposure to CMIT and MIT to levels ranging from at least 4.7-fold to a maximum of 10-fold higher than the RU did not initiate any adverse effects on lung function, morphology, inflammation, and fibrosis. Our findings simulate human exposure conditions and show a lack of effect of Kathon CMIT and MIT on pulmonary tissue. It is concluded that fibrosis initiated by using intratracheal and intranasal instillation is not reflective of human exposure and thus cannot be extrapolated to humans.

## Data Availability

Data presented in this study are available on request from the corresponding authors.
